# Immune Evasion Strategies of *Trypanosoma cruzi*

**DOI:** 10.1155/2015/178947

**Published:** 2015-07-09

**Authors:** Ana Flávia Nardy, Célio Geraldo Freire-de-Lima, Alexandre Morrot

**Affiliations:** ^1^Instituto de Microbiologia, Universidade Federal do Rio de Janeiro, 21.941-590 Rio de Janeiro, RJ, Brazil; ^2^Instituto de Biofísica Carlos Chagas Filho, Universidade Federal do Rio de Janeiro, 21.941-590 Rio de Janeiro, RJ, Brazil

## Abstract

Microbes have evolved a diverse range of strategies to subvert the host immune system. The protozoan parasite *Trypanosoma cruzi*, the causative agent of Chagas disease, provides a good example of such adaptations. This parasite targets a broad spectrum of host tissues including both peripheral and central lymphoid tissues. Rapid colonization of the host gives rise to a systemic acute response which the parasite must overcome. The parasite in fact undermines both innate and adaptive immunity. It interferes with the antigen presenting function of dendritic cells via an action on host sialic acid-binding Ig-like lectin receptors. These receptors also induce suppression of CD4^+^ T cells responses, and we presented evidence that the sialylation of parasite-derived mucins is required for the inhibitory effects on CD4 T cells. In this review we highlight the major mechanisms used by *Trypanosoma cruzi* to overcome host immunity and discuss the role of parasite colonization of the central thymic lymphoid tissue in chronic disease.

## 1. Introduction

*Trypanosoma cruzi *(*T. cruzi*) is a protozoan parasite that causes Chagas disease, which affects nearly 20 million people in the Americas [1, 2]. The disease progresses to a symptomatic chronic phase in which about 30% of patients develop cardiomyopathy or neuropathies and dilatations of the colon or esophagus at some time during their lifetimes [3]. Distinct hypotheses have been considered for the pathogenesis of Chagas disease, including autoimmune effects and parasite-driven tissue damage [4–6].* T. cruzi* is a hemoflagellate parasite with a complex life cycle in which it enters vertebrates through the bite of a haematophagous triatomine (reduviid) insect. The life cycle has distinct stages involving epimastigotes and metacyclic trypomastigotes in the insect vector and blood-form trypomastigotes and intracellular amastigotes in vertebrate hosts [7]. In vertebrates, the parasite confronts a sophisticated immune system involving circulating cells and molecules as well as specialized tissues and organs [8–12].

To overcome host immunity, the trypanosome has an arsenal of evasion strategies linked to alternation between intracellular proliferative forms and nonproliferative, infective extracellular trypomastigotes. The different morphological life cycle forms are associated with adaptive changes in gene expression [13]. Genomic analysis has predicted the protein-coding sequences of* T. cruzi *and has annotated gene clusters/virulence factors implicated in evading host cell immunity. These factors are responsible for the wide range of host cells targeted by the parasite, mainly nonphagocytic cells [14]. Immune evasion by* T. cruzi *relies primarily on subverting the complement system and inhibitory effects on the mononuclear phagocyte system [15, 16].

Downregulation of phagocytic activity is also seen in other protozoan infections such as leishmaniasis and African trypanosomiasis, pointing to evolutionary convergence in the phylogeny of the protozoan parasites [17–19]. However, in contrast to other protozoan parasites that inhibit the maturation of phagolysosomes,* T. cruzi *evades macrophage microbicidal activity by escaping from the phagolysosome to the host cell cytoplasm where it replicates [20]. Moreover, it also interferes with the transcription of cytokines secreted by infected macrophages [21]. TLR activation by the parasite is weak and a major parasite cysteine-protease prevents macrophage activation by blocking the NF-*κ*B P65 pathway and shutting down the express ion of the proinflammatory cytokine, IL-12 [22]. In this scenario, the infection of macrophages favors the secretion of anti-inflammatory cytokines such as IL-10 and TGF-*β* that impair the development of protective immune responses and favor the spread of infection [23, 24].

However, there are a variety of natural strains of* T. cruzi *and it appears that their immune modulatory effects are strain-dependent, a feature that may influence parasite-host interactions [25]. Phylogenetic reconstructions by comparative analysis of the RNA sequences of the various strains suggest that they diverged about 100 million years ago [26]. The different strains coexist dynamically in natural reservoirs. In fact, different combinations of* T. cruzi *strains have been found in the triatomine bugs from domestic and peridomestic areas [27]. Moreover, immune evasion may occur at the population level rather than at the level of a single strain. The CD8^+^ T cell immunodominant epitopes encoded by the large trans-sialidase family of genes vary depending on the parasite strain [28]. As CD8^+^ T cells are crucial for controlling the intracellular parasite, the T cell-mediated cytotoxic mechanisms that prevent parasite growth inside the host also vary.

## 2. Parasite-Associated Acute Phase Virulence Factors Can Overcome the Host Resistance Mechanisms and Establish Persistent Infections

The acute phase of Chagas disease is characterized by strong inhibition of the host immune response by the* T. cruzi *virulence factors, which are crucial for creating a persistent infection and establishing the chronic disease [5, 29, 30]. In both humans and experimental models, the acute phase is marked by a state of immunosuppression [5, 31–39] involving, among other things, the induction of anergy and clonal deletion in the T cell compartment, together with strong polyclonal B cell stimulation which ultimately restricts the development of antigen-specific lymphocytes [40, 41].

In fact* T. cruzi *provides a striking example of an immunosuppression strategy: thus, T cells from infected mice respond poorly to mitogens [33, 34, 37] and they also undergo enhanced apoptosis when the T cell receptor (TCR) is activated, hence increasing the unresponsiveness of host immunity [42–44].* T. cruzi *membrane glycoproteins are critical for damping host protective immunity. The parasite surface is covered by mucin-like molecules with, attached to their terminal *β*-galactosyl residues, sialic acid residues which are transferred from host glycoconjugates by the parasite trans-sialidase [45–48]. These* T. cruzi *mucins are O-glycosylated Thr/Ser/Pro-rich proteins; they are the predominant glycoproteins on the parasite surface and are encoded by more than 800 genes comprising approximately 1% of the parasite genome [49–51].

The* T. cruzi *mucin-like molecules are key players in the host-parasite interplay, including invasion of the host and subversion of its immune system. Their sialylated forms are able to protect parasite antigenic determinants from host attack mediated by anti-galactosyl antibodies and complement factor B [52–55]. They also impair host dendritic cell function as demonstrated by inhibition of the production of IL-12 [56]. This inhibition may occur at the transcriptional level, since the* T. cruzi*-derived mucins are able to inhibit transcription of the IL-2 gene in T cells [33, 34], which also occurs when T cell activation and proliferation are blocked in response to mitogens and antigens [57]. The parasite sialoglycoproteins also inhibit early events in T cell activation, in particular tyrosine phosphorylation of the adapter protein SLP-76 and the tyrosine kinase ZAP-70 [37].

We have recently examined the inhibitory effects of the* T. cruzi *mucins* in vivo*. After exposure to these mucins during experimental infection in a murine model of Chagas disease, the mice displayed increased susceptibility to infection, with enhanced parasitemia and heart damage. These effects were associated with a reduction in IFN-*γ*-producing CD4^+^ and CD8^+^ T cell responses, together with decreased levels of both splenic IFN-*γ* and TNF-*α* [57]. With regard to the molecular mechanisms underlying these effects it has been shown that parasite-derived mucins bind to the mammalian acid-binding Tg-like lectin, Siglec-E (CD33) [58, 59], and our data suggest that binding of Siglec-E by* T. cruzi *mucins inhibits mitogenic responses in CD4^+^ T cells. We showed that the impairment of TCR/CD3-mediated activation of CD4^+^ T cells was correlated with arrest in the G1/S transition of the cell cycle and that interaction of the terminal sialyl residues of the* T. cruzi *mucins with CD4^+^ T cells led to the induction of p27/Kip1, a cell cycle regulator that blocks the transition from G1 to S phase of the cell cycle [57].

The limited T cell responses contrast with the extensive polyclonal expansion of B cell lymphocytes in the acute phase of Chagas disease [41]. During infection an increased frequency of IgG2a- and IgG2b-secreting B cells can be observed in peripheral lymphoid organs. The majority of these cells are nonspecific and secrete antibodies with low affinity for* T. cruzi *antigens [60]; some cross-react with heart and neural tissue [61–63]. These autoantibodies are believed to play secondary roles in the pathogenesis of Chagas disease; they do not induce autoimmune effects because negative selection in the thymus during the process of central tolerance creates a peripheral lymphocyte repertoire with low affinity for cross-reacting autoantigens [64–66].

However, the polyclonal activation of the B cell compartment could restrict the size of the niche needed for optimal development of antigen-specific lymphocytes involved in protective responses to the infection by increasing competition for activation and survival signals in the lymphoid tissues [67, 68]. This phenomenon could have a role in the immunosuppression seen in both mice and humans in the acute phase of Chagas disease [5, 32–39]. Alteration of the homeostasis of the B cell compartment by the parasite has been attributed, at least in part, to parasite-derived glycoinositolphospholipids (GIPLs) [69], which are components of the dense glycolipid layer covering the parasite cell surface [70]. These GIPLs act as virulence factors that function as TLR4 agonists with proinflammatory effect [71]. There is also evidence that the proline racemase encoded by* T. cruzi*, which participates in arginine and proline metabolism, functions as a potent mitogen for B cells and may therefore play a role in immune evasion by the parasite and its persistence in the vertebrate host [72, 73].

## 3. The Impact of Central Tolerance of Parasite-Specific T Cells Targeting the Thymus on Persistent Infection in Chronic Chagas Disease

Pathogens are able to interfere with vertebrate homeostasis at several levels. One important level involves the intersection between the three regulatory systems, neural, endocrine, and immune. These physiological networks can work together to recognize the danger of pathogen invasion. In most vertebrates threatened by a pathogen, acute short-term stress signals induce host responses that enhance innate defense mechanism [74]. A race between the pathogen-mediated evasion mechanisms and host immune responses then determines whether the invader will be rapidly eliminated or establish a persistent infection. In the latter case, the stress signals continue to suppress the host immune response, a scenario that favors the infection. It has been shown, for instance, that chronic stress causes a shift from T helper 1-mediated cellular immunity towards T helper 2-mediated humoral immunity, and this can influence the course of an infection and the susceptibility of the host to intracellular pathogens [75].

In infections caused by* T. cruzi*, TNF-*α* induces an inflammatory syndrome during the acute phase which activates the hypothalamus-pituitary-adrenal (HPA) axis leading to release of corticosterone. This stress hormone affects the disease outcome by its effect on the host immune system [76, 77]. Endogenous glucocorticoids also have an impact on the thymus, the central lymphoid organ controlling the continuous formation of T cells which are released to the periphery to form the host immunological repertoire [78]. The complex developmental processes in the thymus depend on direct contact between stromal cells and the thymocytes undergoing maturation, and disturbance of the thymic microenvironment can affect the T cell repertoire and thus the adaptive immune response [79].

Alterations of the thymic environment occur in infections involving many distinct pathogens (bacteria, viruses, parasites, and fungi) [80–88]. In most cases, disruption of thymic homeostasis can cause atrophy of the organ due to the apoptotic death of thymocytes [79]. This is the case in experimental models of* T. cruzi *infection, in which an imbalance between intrathymic and systemic stress-related endocrine circuits gives rise to high levels of intrathymic glucocorticoid hormones that mainly affect the viability of CD4^+^CD8^+^ thymocytes, but the populations of other subtypes such as double-negative (DN) T cells and SP cells are also reduced [76, 89]. This death mechanism is associated with the activation of thymocyte caspases 8 and 9, which promote apoptotic cell death [90].

Another contribution to thymic atrophy in Chagas disease is the premature export of immature thymocytes to the periphery. We have shown that the infection results in premature release of immature CD4^−^CD8^−^ double-negative thymocytes, as well as CD4^+^CD8^+^ double-positive thymocytes that have a proinflammatory activation profile [91–93]. We also found elevated levels of undifferentiated T lymphocytes in the peripheral blood of patients with severe cardiac forms of chronic Chagas disease and obtained evidence that the migration of very immature thymocytes from the infected thymus is due to sphingosine-1-phosphate receptor-1-dependent chemotaxis [94]; this points to an important role for sphingolipid signaling in the escape of undifferentiated thymocytes to the periphery in Chagas disease.

Although many pathogens induce thymic atrophy, until recently it was not clear whether negative selection eliminating T cells bearing TCRs against self-antigens was affected in the atrophic thymus. We answered this question by showing that the expression of peripheral antigens in the infected thymus is sufficient to promote negative selection in tolerance induction [93]. We provided evidence that immature thymocytes undergoing intrathymic maturation can be negatively selected during thymic atrophy. This corroborates the evidence that mature single-positive CD4^+^ and CD8^+^ T cells exiting the thymus do not harbor forbidden TCR genes [93, 95].

Although our data strengthen the notion that the infected thymus undergoing atrophy is still able to carry out negative selection, this matter should be thought of in the context of host-pathogen interactions. In Chagas infections, the parasites can colonize the thymus [96]. As a consequence, their antigens may be presented to recycling memory parasite-specific T cells moving from the periphery to the thymic microenvironment. The activation of these cells within the thymus could render them susceptible to the process of clonal deletion promoted by the thymic recognition of cognate antigens.

In addition, there is an alternative pathway in the thymus leading to the development of regulatory T cells recognizing specific antigens with high affinity TCRs [97]. Hence, the presence of* T. cruzi *antigens in the thymus could lead to the generation of parasite-specific regulatory T cells contributing to tolerance to parasites that target the thymus. If that process in fact occurs, it could induce central tolerance to the parasite antigens, thus undermining the establishment of protective immunity during the course of chronic disease. These issues are relevant to all host-pathogen interactions involving the thymus.

## 4. Concluding Remarks

The protozoa are the most ancient members of the animal kingdom and they have evolved to become one of the most dominant forms of life on earth. This evolutionary branch gave rise to the* Trypanosome cruzi*, a member of the kinetoplastid protozoa [98]. The survival of these parasitic unicellular organisms to the present day owes much to their efficient reproductive mechanism, with its short generation times and rapid developmental sequence producing large numbers of progeny [27]. These attributes lead to powerful infections, with an acute phase that strongly activates the host immune response. However, the acute phase involves complex molecular and cellular interactions between the pathogen and its host that can be exploited to the parasite's benefit [5, 29, 30].

The systematic study of experimental* T. cruzi *infection models reveals that the parasite has ways to subverting immune defenses, and genomic studies have disclosed that it has evolved many genes devoted to this purpose [99]. However, important recent investigations focus on a new aspect of the parasite's evasion mechanism that may favor its chronic persistence in the host. These studies have shown that different parasite strains coexist in their natural reservoirs [27]. This creates a situation where the dynamics of antigen variation within a parasite population expressing distinct subdominant T cell epitopes with low binding affinity to major histocompatibility complex (MHC) molecules could subvert host adaptive immune responses.

In addition, our studies have raised important questions about how the parasite undermines the host immune system at a more profound level and so increases its chance of persisting chronically. The finding that the parasite targets the thymus but does not alter the capacity of the organ to induce clonal deletion of antigen-specific T cells highlights the relevant issues to be approached [93]. It is reasonable to consider that the presence of pathogen antigens in the thymus may induce the recirculation of activated T cells from the periphery to the thymus in an attempt to prevent colonization of the organ. This scenario may induce clonal deletion of pathogen-specific T cells that recognize antigens via thymic dendritic cells involved in negative selection. Alternatively, it could lead to the generation of pathogen-specific regulatory T cells that induce tolerance to persistent infection. These possibilities are important for our understanding of the establishment of T cell protective immunity and the host's ability to control chronic persistent pathogen infections.
